# Photoacoustic Imaging to Track Magnetic-manipulated Micro-Robots in Deep Tissue

**DOI:** 10.3390/s20102816

**Published:** 2020-05-15

**Authors:** Yan Yan, Wuming Jing, Mohammad Mehrmohammadi

**Affiliations:** 1 Department of Biomedical Engineering, Wayne State University, Detroit, MI 48201, USA; yyan2@wayne.edu; 2 A. Linton Department of Mechanical Engineering, Lawrence Technological University, Southfield, MI 48075, USA; wjing@ltu.edu

**Keywords:** photoacoustic, ultrasound, micro-scale, microrobot, magnetic

## Abstract

The next generation of intelligent robotic systems has been envisioned as micro-scale mobile and externally controllable robots. Visualization of such small size microrobots to track their motion in nontransparent medium such as human tissue remains a major challenge, limiting translation into clinical applications. Herein, we present a novel, non-invasive, real-time imaging method by integrating ultrasound (US) and photoacoustic (PA) imaging modalities for tracking and detecting the motion of a single microrobot in deep biological tissue. We developed and evaluated a prototyped PA-guided magnetic microrobot tracking system. The microrobots are fabricated using photoresist mixed with nickel (Ni) particles. The microrobot motion was controlled using an externally applied magnetic field. Our experimental results evaluated the capabilities of PA imaging in visualizing and tracking microrobots in opaque tissue and tissue-mimicking phantoms. The results also demonstrate the ability of PA imaging in detecting a microrobot with the sizes less than the minimum detectable size by US imaging (down to 50 µm). The spectroscopic PA imaging studies determined an optimal wavelength (700 nm) for imaging microrobots with embedded Ni particles in oxygenated (fresh) human blood. In addition, we examined the ability of PA imaging to detect the microrobots through a nontransparent tissue mimic and at a depth of 25 mm, where conventional optical methods are unable to be used in tracking the objects. These initial results demonstrate the feasibility of an integrated US and PA imaging method to push the boundaries of microrobot applications into translational applications.

## 1. Introduction

Microscopic, untethered, mobile robots have been envisioned as one of the next-generation intelligent robotic systems [1]. The potential biomedical applications of microrobots are aimed towards non-invasive operations in circulatory, urinary, and central nervous systems [2,3]. However, the visualization and accessibility of microrobots in clinical conditions are the major difficulties preventing the use of microrobots in biomedical applications. Currently, optical (i.e., camera) and ultrasonic (B-mode ultrasound) [4,5,6] modalities have been used to track the motion of microrobots during their operation. The requirement of a transparent imaging path (direct optical access to the microrobots) for optical-camera based detection systems violates the purpose of non-invasive operation. In contrast, ultrasound (US) imaging is introduced as an alternative non-invasive visualization modality [7]. However, clinical US imaging has a tradeoff between its resolution and penetration depth, which limits the ability of the US to provide accurate and high contrast images of small-sized microrobots [8]. Previous studies have determined the capability of US imaging when detecting micro-scale microrobot motion in turbid tissues [4,9]. However, these visualization techniques were either based on the bubbles emitted from the microjets [9] or a massive swarm of agents [4], which were both at the millimeter scale. The ability to detect micro-scale microrobots in turbid tissues poses a challenge for current visualization technologies. An alternative imaging tool to overcome these limitations, i.e., the penetration depth of optical imaging and low sensitivity and accuracy of US imaging, can play an important role in the translation of microrobot technologies to pre-clinical and clinical applications.

Photoacoustic (PA) imaging is a relatively new biomedical imaging modality [10,11,12] and has shown tremendous potential in diagnostic applications by providing complementary functional and molecular information [13,14,15,16,17,18,19,20,21,22,23]. PA imaging is based on the photoacoustic effect, which transforms the object’s optical properties into broadband acoustic pressure waves. It utilizes a short (typically nanosecond) laser pulse to illuminate the tissue of interest. The tissue chromophores or an exogenous absorber absorb the light energy and release acoustic waves due to rapid thermoelastic expansions. These acoustic waves can be probed to form superimposed USPA images. By using this effect, the micro- to nano-scale objects, which have a size below the minimum detectability of a clinical US imaging system, can be visualized in deep tissue layers. Since PA imaging relies on optical absorption, metallic particles and objects are suitable candidates to be imaged with a high imaging contrast. For example, PA imaging has been utilized in detecting inserted metallic objects such as biopsy needles and brachytherapy seeds [24,25], as well as monitoring drug delivery with microcapsules [26]. PA imaging, in conjunction with the US system, can potentially enhance the sensitivity of detecting and tracking of the microrobots, while US imaging provides a co-registered image of the background tissue. A PA imaging system can be built around a clinical US scanner, since the US and PA share the same acquisition hardware and the image reconstruction method. Integrated USPA imaging is a suitable candidate to increase the sensitivity of microrobots. Herein, we proposed combined US and PA imaging modalities for accurate visualization and enhanced motion sensing of microrobots in turbid, enclosed environments.

## 2. Materials and Methods

### 2.1. Design and Development of PA-Sensitive Magnetic Microrobots

**Figure 1:**
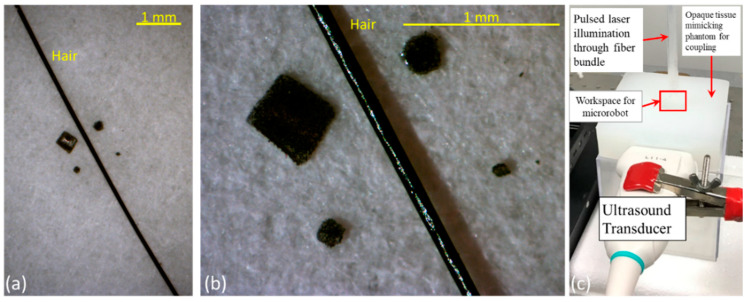
Photograph of the micro-scale microrobots with sizes of 400, 200, 100, and 50 µm at (**a**) no zoom, (**b**) 4× zoom. (**c**) Experimental setup for the integrated USPA imaging system for tracking microrobots. (The US platform, power source for the laser, and magnet are not shown in the figure).

PA imaging is sensitive in detecting photoabsorbers, such as metallic particles. Based on this principle, we developed a prototype of PA-sensitive magnetic, micro-scale robots with Nickel (Ni, Alfa Aesar, Haverhill, MA, USA) particles. The average diameter of the Ni particles is approximately 3–7 µm that fits in the microrobot’s envelope dimension. The Ni particles were mixed in a negative photoresist (SU-8-2035, MicroChem Corp. Westborough, MA, USA) at a density of 1.25 g/mL (volume ratio ≈ 1:7). The sample was then patterned through photolithography to produce microrobot prototypes with different dimensions (400 µm, 200 µm, 100 µm, 50 µm square-shaped, as shown in Figure 1a,b). This photolithography process produces an undissolvable polymer structure, with temperature stability as high as 150 degrees Celsius, which will prevent the leaching of Ni particles and will avoid contamination from Ni particles. While we used polymer-based constructs in this study, other strategies such as silica coating [27,28] can be utilized to ensure the thermal stability and reduce microrobot cytotoxicity concerns. The thickness of the developed microrobots is approximately 40 µm. The 99.9% basic metal particles and dark grey to black color provide strong optical absorption, which generates a large PA imaging contrast.

### 2.2. Integrated Ultrasound and Photoacoustic (USPA) Imaging System

The integrated USPA imaging system combines the advantages of both US and PA imaging. The US imaging visualizes the anatomical and the structural information of the tissue, while PA imaging increases the sensitivity of detecting micro-scale objects. The US and PA imaging setup is shown in Figure 1c. The system consists of two main parts, acquisition and illumination sections. The acquisition system utilizes a clinical linear US probe (L11-4v, Philips Inc. Ville Platte, LA, USA) that connects to a 128 channel US research platform (Vantage 128, Verasonics Inc. Kirkland, WA, USA). The US probe, operating at 11 MHz, provides a theoretical axial resolution of about 120 µm. This theoretical resolution limits the US imaging to accurately detecting objects with sizes below 120 µm. In other words, the accuracy of US imaging in locating and tracking smaller microrobots is determined by the spatial resolution of US imaging. On the contrary, PA imaging is capable of detecting smaller microrobots due to the broader bandwidth acoustic waves generated from the object. The illumination component consists of a tunable, high energy, short-pulsed laser (OPOTEK core, 650 nm to 2400 nm, 10 Hz, eight ns pulse duration, Opoteck Inc. Carlsbad, CA, USA) and a fiber optic light delivery. The laser beam was coupled to a 19-fiber bundle (FT1000EMT, Thorlabs Inc. Newton, NJ, USA), in which the proximal end was bundled, and the distal end was kept free. During the experiments, the distal end of 18 fibers was packed into a 5 mm diameter illumination pattern targeting the imaging workspace. The 19th fiber was used to monitor the real-time laser energy during experiments. The integrated USPA imaging system is capable of providing real-time, high resolution, and co-registered US and PA images. 

In traditional optical-based microrobot visualization systems, the workspace is composed of an optically transparent material, which does not mimic soft biological tissues. During our experiments, we made two imaging phantoms that provided enclosed workspaces, while at the same time coupling the acoustic waves arising from the objects. These phantoms have relatively similar mechanical and acoustic properties to soft tissue and allow for the transmission of US and PA pressure waves from and to the US transducer. The two workspaces involved in this study are comprised of (1) polyvinyl alcohol (PVA) and (2) polyvinyl chloride (PVC). The PVA workspace was made of 10% (wt/water) PVA. The overall phantom dimensions were 10 × 10 × 5 cm^3^, and the center of the phantom contained a 2×2×1 cm^3^ enclosed workspace for microrobots. The top of this enclosed workspace was covered with removable plastic material to act as an optical window. The optical images of the microrobot in this workspace were acquired during each experiment. The PVC phantom measured 5×5×7.5 cm^3^ with a 1×1×7.5 cm^3^ square-shaped, see-through hole at its center. The bottom and the top of the PVC phantom were enclosed with plastic covers. During the experiments, an optical camera was placed at the bottom of the phantom to record the motion of the microrobot as a reference. 

### 2.3. USPA Imaging for Detecting Microrobots during Static and Variable Motion

**Figure 2:**
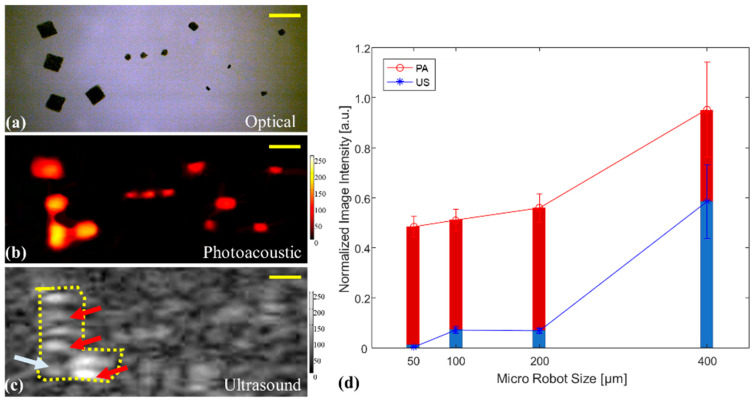
Imaging result of static microrobots with different sizes in a pattern of “L•••W.” (**a**) Reference photo through the optical camera in an open workspace. (**b**) The detected PA image in an enclosed workspace. (**c**) Imaging result of the US method. The red arrows indicate the US artifacts, and the white arrow shows the microrobot was not detected in the US. (**d**) Imaging intensity analysis for US and PA methods of detecting varied dimensions of microrobots. The scale bars are 1 mm.

Experiments were performed to evaluate the sensitivity of the proposed integrated USPA imaging method in detecting the motion of microrobots in static and dynamic environments. Twelve microrobots of different sizes (from 400 to 50 μm) were placed at the bottom of the PVA workspace in the static case to form a specific pattern depicting the two initial letters of collaborating universities, “L···W” (Figure 2a). The “L” included four 400 μm microrobots; the “···” was formed by three 100 μm microrobots. The top two dots in the “W” were 200 μm, and the bottom three dots for W were 50 μm. The PVA workspace was filled with distilled (DI) water to enable acoustic coupling for USPA imaging. The contrast to noise ratio (CNR) and signal to noise ratio (SNR) were calculated to quantify the performance of USPA imaging for detecting microrobots of different dimensions. The regions of interest (ROIs) for CNR and SNR calculations were selected based on the optical image. SNR and CNR values were calculated as follows:

(1) $$SNR=20\log\left(\frac{mean\left(Obj\right)}{std\left(Bkgnd\right)}\right)$$

(2) $$CNR=20\log\left(\frac{mean\left(Obj\right)-mean\left(Bkgnd\right)}{std\left(Bkgnd\right)}\right)$$

where Obj indicates the PA signal from microrobot objects within the selected ROI(s), and Bkgnd is the PA values of the pixels located within the background. Mean and std represent the average and standard deviation, respectively.

**Figure 3:**
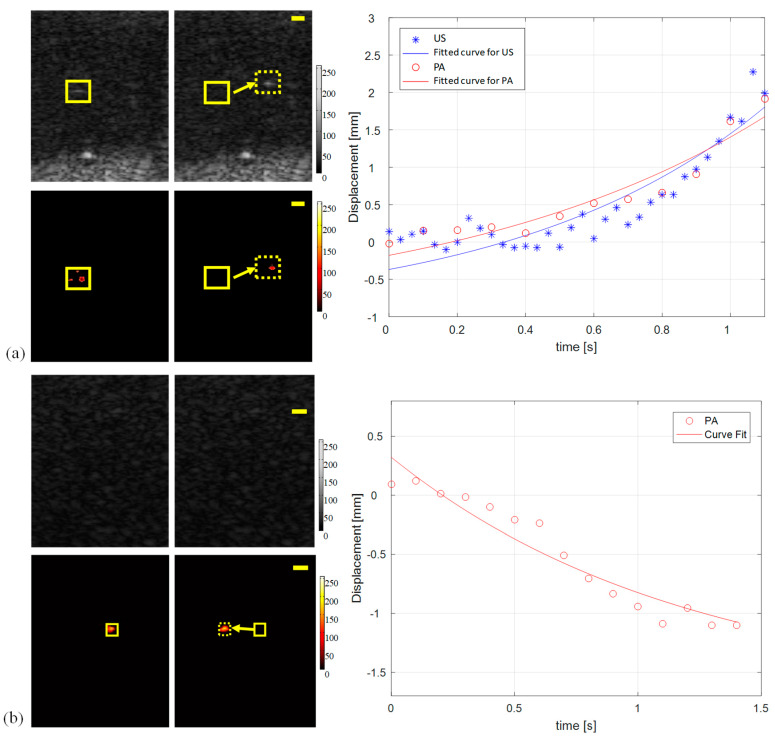
US and PA tracking results of a single 400 µm microrobot (**a**) and 100 µm microrobot (**b**) in motion. The imaging results indicated the PA imaging has higher sensitivity to detect the smaller microrobot, where the US was unable to track the object. The solid rectangles in the images demonstrate the start position of the microrobot, and the end positions were marked as dashed rectangles. The solid lines in the fitted curves estimate the motion of the microrobot. The scale bars are 1 mm. The color bar shows the image intensity.

In theory, microrobots with sizes between 400 and 200 μm can be visualized in both US and PA images. Nevertheless, US imaging is unable to accurately detect microrobots with a size less than 120 μm. In contrast, PA imaging can detect photo-absorbers (i.e., microrobots) with smaller sizes than the US detection limit. The integrated USPA imaging system described above has a temporal resolution of PA and US imaging of 10 Hz and 30 Hz, respectively. The major limiting factor for the lower temporal resolution of PA imaging is the pulse repetition rate of the laser. However, it is possible to increase the laser repletion rate to several hundreds of pulses per second to achieve a faster PA imaging system [29]. The motion of the microrobot was driven by a static magnetic field. During each motion tracking experiment, the PVA workspace contained a single microrobot (Figure 3). The manipulator magnet was kept 15 mm away from the microrobot. As described above, we can estimate that the 400 μm microrobots can be tracked using both US and PA imaging, but the smaller microrobot can only be visualized using PA imaging. By measuring the motion of the magnetic microrobots and through inverse modeling, it is possible to calculate the strength of the manipulating magnetic field (B). The acting magnetic force $F_{m}$ is evaluated as:

(3) $$F_{m}=\nabla \left(m\cdot B\right)=m\cdot \nabla B$$

where m is the magnetization of the nickel material that is treated as a constant. The gradient of a magnetic field, ∇B, along the acting direction, is the other value to be evaluated to derive the acting magnetic force. The magnetization m is estimated through $m=M\cdot m_{Ni}$, where M is the mass magnetization of the nickel material, 55.1 $A\cdot m^{2}\;\cdot {\mathrm{kg}}^{-1}$, and $m_{Ni}$ is the nickel mass embedded in a single microrobot prototype. Take the 400 µm size prototype as an example, $m_{Ni}=400\times 400\times 40{\;\mu m}^{3}\times 1.25\;g/\mathrm{mL}\;=\;8.0\times 10^{-9}\;\mathrm{kg}$. Thus, the magnetization m of this magnetic microrobot is evaluated as $4.4\times 10^{-7}\;A\cdot m^{2}$. The magnetic field is produced by a cone array of multiple permanent magnets fixed at a distance of 15 mm facing the microrobot. 

**Figure 4:**
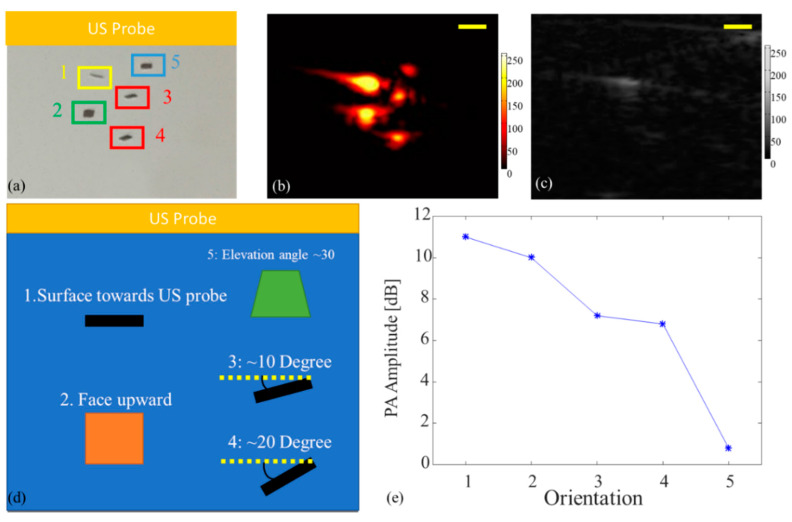
Imaging results for detecting microrobots in different orientations (**a**) Reference photograph, object “1” is parallel, and object “2” is perpendicular to the US probe surface; (**b**) PA imaging visualizes all five objects. (**c**) US imaging only detected object “1”. (**d**) Schematic of the orientation of microrobots. (**e**) Variation of PA signal amplitude at different orientations. The scale bars are 1 mm.

The orientation of the microrobot plays an important role in external controlling via magnets. In our previous study, the angle-independent capabilities of PA imaging were demonstrated [30]. To further extend the findings and to quantify the capability of PA imaging in indicating the orientation of microrobot, a controlled orientation experiment was performed in this study. Five 400 µm sized microrobots were placed in different orientations of the acoustic imaging plane within the workspace of the PVA phantom (Figure 4a). A transparent, high-viscosity liquid (3659-12 Purell, GOJO Industries, Inc. Akron, OH, USA) was used as the medium to limit the motion of the microrobots and maintain their orientations. This allowed for generating a uniform illumination pattern over the microrobots’ workspace and prevented signal changes due to fluence variations. The laser illumination was directed from the top of the PVC containing phantom, which was perpendicular to the US probe. The orientations of the microrobots were set as: (1) the largest cross-section of the microrobot pointing towards the US probe; (2) the largest cross-section placed perpendicular to the US probe; (3) the largest cross-section facing the US probe tilted at an angle of 10 degrees, (4) 20 degrees counterclockwise; (5) the largest cross-section tilting (elevational) at an angle of 30 degrees towards the US probe (Figure 4d). The correlation between the PA signal amplitude to orientation angles was calculated to quantify the capability of PA imaging to detect the orientation of microrobots. 

### 2.4. USPA Imaging of Microrobots through Biological Tissue and Inside Human Blood

**Figure 5:**
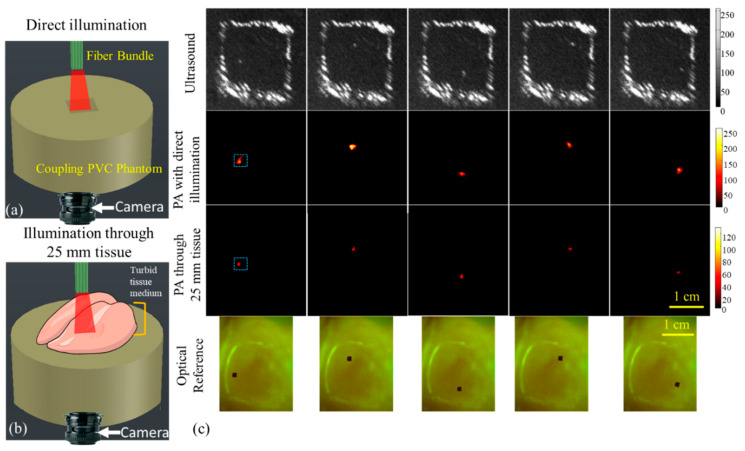
Imaging setup for detecting microrobots with (**a**) direct illumination and (**b**) illumination after a 25 mm chicken breast tissue, and (**c**) US images (top row), PA images with direct illumination (second row), PA imaging through opaque tissue (third row), and optical images (fourth row).

Non-invasive microrobot biomedical applications require the accurate detection of microrobot motion in turbid medium (through tissue layer and blood), as would be the case for operation in the human circulatory system. The nontransparent imaging medium has created significant challenges for the current optical-based guiding methods. The integrated USPA imaging method overcomes the challenges posed by the nontransparent imaging path. The accuracy of motion tracking with USPA imaging was evaluated through a set of ex-vivo tissue experiments. In the experiment, a 25 mm thick chicken breast mimicked the nontransparent imaging path. During the experiment, the bottom side of the see-through hole in the PVC phantom was covered by a thin layer of optically transparent glass. This enclosed workspace was then filled with DI water that created an optical path for the optical camera for viewing the microrobot from the bottom. One 400 μm microrobot was suspended floating at the center of the see-through hole of the PVC phantom. The location of the microrobot was varied through a static magnetic field. At each location, one US image, two PA images, and one optical image were acquired. The two PA images were portrayed with two different illumination strategies (Figure 5a,b): (1) direct illumination (without tissue placed on the path); (2) an illumination through the 25 mm opaque chicken breast tissue. The selected wavelength for PA imaging was 700 nm. The SNR of PA images were compared to demonstrate the feasibility of the USPA system in imaging the microrobot in tissues. The fluence emerging from the chicken breast tissue was significantly decreased due to the highly diffusive nature of the tissues. This led to the change in the PA signal amplitude generated from the microrobots. The detectability of microrobots in these two scenarios was compared through the SNR factor, where a higher SNR represents an enhanced detectability.

**Figure 6:**
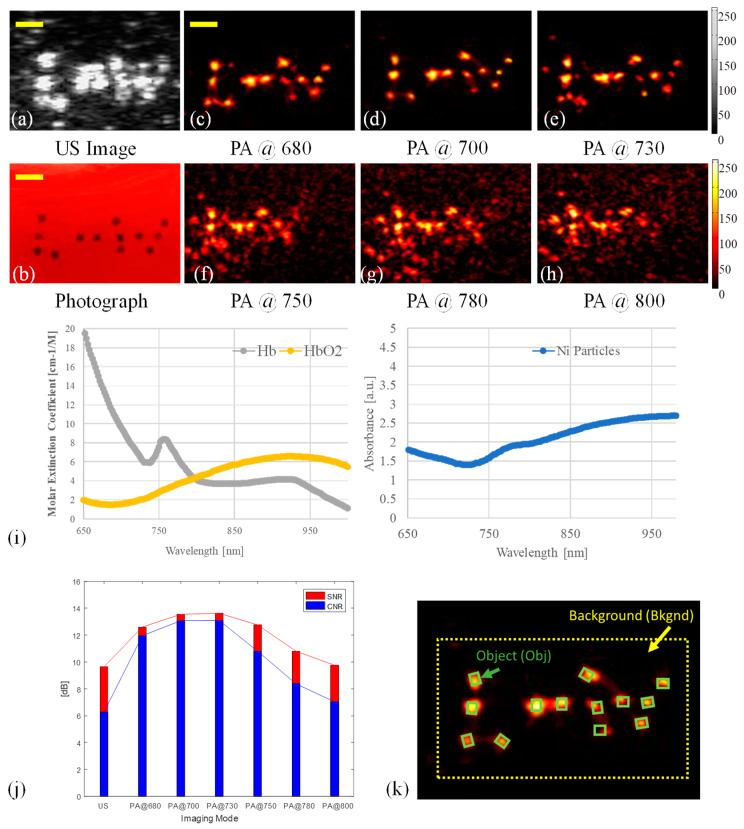
Imaging results for detecting microrobots in human blood. (**a**) US image of the “L··W” pattern. (**b**) Photograph of the pattern before covering with human blood. (**c-h**) PA images at different wavelengths between 680 and 800 nm. (**i**) Human oxy- and deoxy- hemoglobin molar extinction coefficients, and the Ni particle absorbance; the data were adopted from [31] and [32]. (**j**) Calculated SNR and CNR for US and PA images, the ROIs of the objects were selected based on the optical image. (**k**) A sample PA image (acquired at 700 nm) of the selection for ROIs, where the green rectangles indicate the PA signal from microrobot objects, and the yellow rectangle indicates the area of the background. The scale bars are 1 mm.

In addition, another set of ex-vivo experiments were performed to determine the optimized PA imaging wavelength for microrobot detection in human blood. The imaging window for deep tissue PA imaging lies in the range varying from 600 to 900 nm. In this spectral range, the absorptions and scattering of oxyhemoglobin are low compared to Ni particles [31,32]. In this experiment, 13 microrobots with a size of 400 μm were utilized to form a pattern of “L··W” (two dots between “L” and “W”). The pattern was fixed at the bottom of the PVA workspace (Figure 6b). The microrobots were suspended by embedding the pattern at the surface of a gelatin block (2×2×0.5 cm^3^) composed of 10% human blood, 10% porcine skin gelatin, and DI water (wt/wt/wt). The workspace was then filled with 100% human blood to a depth of 5 mm. A set of spectroscopic PA images was acquired at wavelengths varying from 650 to 850 nm with a step size of 10 nm. The optimized PA imaging wavelength in the blood can be determined by calculating the SNR and CNR of the PA images. The selection of objects and background areas was based on the optical images obtained by the camera (Figure 6k). 

## 3. Results and Discussion

### 3.1. USPA Imaging for Detecting Stationary and Moving Microrobots 

Figure 2 shows the results for PA imaging of different sizes of microrobots. These images demonstrated the advantage of PA imaging compared to the US method. PA imaging provides a high-contrast and almost background-free image of the objects due to a large optical contrast between microrobots and the background. A noticeable clearer detection of the “L···W” pattern in PA images compared to the US imaging demonstrates the higher sensitivity of PA imaging when observing micro-scale objects. The PA signal amplitudes also decreased with the reduction of microrobot sizes. The SNR of the PA image was significantly higher than that of the US image (SNR_PA_: SNR_US_ = 55:1). The significant differences are the result of some US reverberation artifacts (red markers) and the large background signal in US images. The microrobot at the corner of the “L” shape was not well visualized by the US imaging system. The US image also had a low CNR and was only able to detect the largest microrobots in the “L” shape while the smaller sizes were invisible. In contrast, the PA imaging was able to detect all sizes of the microrobot prototypes with the lowest CNR of 22 dB.

The microrobot motion tracking results using USPA imaging are shown in Figure 3. The left panel shows the US and PA images for the start and end positions of the moving microrobot. As expected, the US and PA imaging successfully tracked the movement of the largest microrobot. The smaller microrobot was only visualized by PA imaging. Based on the graphs depicted in the right panel and Equation (1), we demonstrated that the average acceleration of the 400 μm microrobots is 3.3 m/s^2^ and the sum driving force on it is approximately $4.2\times 10^{-11}\;N,$ which is the same magnitude as the estimation of the acting magnetic force. For a 100 μm sized microrobot, the acceleration is 2.1 mm/s^2^, and the average driving force is assessed as $1.7\times 10^{-12}\;N$, which also has the same magnitude. The average speed of microrobots was approximately 2.10 mm/s and 1.14 mm/s during the two imaging tests, which correspond to about 5 and 12 body-length/s, respectively. The imaging results indicate that the current PA imaging temporal resolution is sufficient to track the microrobot movement in real-time. The low frame rate of PA imaging can be improved by using a higher repetition rate laser source and can reach up to several thousands of frames per second (fps). The detected motion shown in Figure 3a was acquired with a 10 Hz PA frame rate and a 30 Hz US frame rate, showing maximum differences below 5 percent. The better sensitivity of PA imaging may be mainly due to the inaccurate visualization of the objects with US imaging. Besides, as shown in Figure 3b, the US imaging cannot detect the smaller microrobot prototype. In contrast, the PA imaging method can still track the microrobot. The experimental results also indicate that the motion of the microrobots due to the manipulating magnetic field is not inertia dominated. The micro-scale object was mainly affected by forces such as fluid tension/drag displacement over time. From the above tests, the tracking capacity of USPA imaging for a single micro-scale mobile microrobot is verified. Moreover, the USPA imaging modality also introduces better tracking sensitivity than the US imaging method.

During the motion of microrobots in a fluid environment, the microrobot experiences both translational and rotational motion. Imaging microrobots at different orientations poses a challenge for US imaging because the object with a tilted angle reflects the acoustic wave out of the receiving plane and may not be properly visualized. However, PA imaging does not suffer from angular dependency since the generated PA signal travels omnidirectionally and thus is independent of the orientation. As shown in Figure 4b,c, the microrobots with large orientation angles with respect to the US probe surface were not detected by US imaging. In contrast, PA imaging accurately detected all five microrobots. The calculated PA signal amplitudes (Figure 4e) also showed a relation between the efficient illumination area of the microrobot and its surface area towards the US transducer. It is anticipated that the highest PA amplitude was from orientation “2” since it has the largest optical absorption cross-section (with illumination being from the top of the workspace and perpendicular to the US and PA imaging planes). However, the large amplitude of the PA signal was detected at orientation “1”. We believe that the amplitude of the PA signal strength, detected at the transducer location, will depend on both the initial PA pressure (proportional to the effective surface area) and the location/orientation of the acoustic detector. The latter is due to the microrobot geometry, which creates a level of “directionality” to the PA signal propagation. In other words, we think that the spatial propagation PA signal generated by microrobots is different from a spherical wave from a point source. Therefore, the amplitude of the PA signal received from the microrobot facing the transducer is strong since the larger portion of the generated PA signal is propagated towards the transducer. To better understand the relation between the PA signal and the orientation of microrobots, conducting simulation studies is within the scope of our future studies.

### 3.2. PA Imaging of Microrobots through Opaque Tissue and in Human Blood

The experimental results indicating the capability of the USPA imaging method in detecting the microrobot in deep tissue are shown in Figure 5. Two illumination strategies are utilized in this experiment: (1) direct illumination (Figure 5a) and (2) light passing through a 25 mm thick chicken breast tissue (Figure 5b). The US, PA, and optical images of the microrobot detected at different locations are shown in Figure 5c. These results show the ability of USPA to detect the location of the microrobot through a thick layer of opaque tissue. We also have demonstrated the capability of PA imaging in detecting microrobots through a nontransparent medium and at an imaging depth of 25 mm. As expected, the PA signals were reduced (by 70%) when the microrobots were imaged using an illumination through the chicken breast tissue and due to the light-diffusing and attenuation. Therefore, we can conclude that the proposed USPA imaging method is suitable for detecting a microrobot in biological tissues. This unique feature of the USPA imaging method will push the boundary of the current microrobot clinical applications.

We explored the feasibility of the integrated USPA imaging modality to track the microrobot in a highly light absorptive medium, such as human blood. An experiment was performed where 400 µm microrobots forming an “L··W” pattern were placed in human blood (Figure 6b). The pattern was successfully imaged by both US and PA imaging. The background noise in the PA images, originating from the surrounding human blood, increased significantly after 730 nm, which matched the elevated absorption of oxygenated hemoglobin. The SNR of PA images was quantified across different wavelengths. The wavelength of 700 nm showed the highest CNR. Since we used oxygenated fresh human blood, the majority of the background PA signal is anticipated to match the absorption spectrum of oxyhemoglobin. In this study, the wavelength of 700 nm, at which HbO_2_ has a low HbO_2_ optical absorption [31], was selected as an optimal imaging wavelength for imaging microrobots through oxygenated human blood. It is necessary to mention that this optimum wavelength may change depending on the medium in which the microrobots are located. Besides the acoustic artifacts such as reverberation, the size of visualized microrobots in the US image was increased 45% more than their actual physical size.

## 4. Conclusions

In this study, PA imaging is introduced as a suitable imaging solution for localizing and tracking magnetic microrobots in tissue-mimicking environments. PA provides a platform for non-invasive tracking and visualizing of micro-scale magnetic microrobots operating in a turbid biological working environment. This technology utilizes PA imaging to visualize the miniaturized microrobot prototype superimposed on US structural images of tissue background. The experimental results have verified the enhanced sensitivity of PA imaging modality compared to traditional US techniques. The experimental results of PA imaging in deep biological tissues and human blood demonstrate the feasibility of utilizing this for potential pre-clinical and clinical applications in which tracking microrobots are required. The non-invasiveness of USPA imaging will enable the translation of this technology into potential clinical applications involving intelligent micro-scale microrobots.
